# Identifying longitudinal clusters of multimorbidity in an urban setting: A population-based cross-sectional study

**DOI:** 10.1016/j.lanepe.2021.100047

**Published:** 2021-03-02

**Authors:** Alessandra Bisquera, Martin Gulliford, Hiten Dodhia, Lesedi Ledwaba-Chapman, Stevo Durbaba, Marina Soley-Bori, Julia Fox-Rushby, Mark Ashworth, Yanzhong Wang

**Affiliations:** a School of Population Health & Environmental Sciences, Faculty of Life Sciences and Medicine, King's College London, London, UK; b NIHR Biomedical Research Centre, Guy's and St Thomas’ NHS Foundation Trust and King's College London, London, UK

**Keywords:** Multimorbidity, Clustering, Correspondence analysis, Long term conditions, LTC, long term conditions, MCA, multiple correspondence analysis, UK, United Kingdom, QOF, quality outcomes framework, WSS/BSS, ratio of within- to between- sum of squares

## Abstract

**Background:**

Globally, there is increasing research on clusters of multimorbidity, but few studies have investigated multimorbidity in urban contexts characterised by a young, multi-ethnic, deprived populations. This study identified clusters of associative multimorbidity in an urban setting.

**Methods:**

This is a population-based retrospective cross-sectional study using electronic health records of all adults aged 18 years and over, registered between April 2005 to May 2020 in general practices in one inner London borough. Multiple correspondence analysis and cluster analysis was used to identify groups of multimorbidity from 32 long-term conditions (LTCs).

**Results:**

The population included 41 general practices with 826,936 patients registered between 2005 and 2020, with mean age 40 (SD15·6) years. The prevalence of multimorbidity was 21% (*n* = 174,881), with the median number of conditions being three and increasing with age. Analysis identified five consistent LTC clusters: 1) anxiety and depression (Ratio of within- to between- sum of squares (WSS/BSS <0·01 to <0·01); 2) heart failure, atrial fibrillation, chronic kidney disease (CKD), chronic heart disease (CHD), stroke/transient ischaemic attack (TIA), peripheral arterial disease (PAD), dementia and osteoporosis (WSS/BSS 0·09 to 0·12); 3) osteoarthritis, cancer, chronic pain, hypertension and diabetes (0·05 to 0·06); 4) chronic liver disease and viral hepatitis (WSS/BSS 0·02 to 0·03); 5) substance dependency, alcohol dependency and HIV (WSS/BSS 0·37 to 0·55).

**Interpretation:**

Mental health problems, pain, and at-risk behaviours leading to cardiovascular diseases are the important clusters identified in this young, urban population.

**Funding:**

Impact on Urban Health, United Kingdom.

**Research in context**

**Evidence before this study**

Two systematic reviews have been published on the clustering of long-term conditions, based on the non-random association between diseases (Prados-Torres A et al. J Clin Epidemiol 2014;67(3):254–66; Busija L et al. Eur J Epidemiol. 2019;34(11):1025–1053). All identified studies used a cross-sectional design, with heterogeneity in the techniques used. Only the latter review included a study within the United Kingdom and this study used a small sample of hospitalized patients aged over 85 years. Recently, Zhu et al. (2020) clustered multimorbid adults in the UK whose diagnoses were defined in 2012. These clusters were based on individuals and not diseases. Relationships were found between psychoactive substance and alcohol misuse in those aged 18–64; coronary heart disease, depression and pain (aged 65–84); and coronary heart disease, heart failure and atrial fibrillation (aged 85+).

**Added value of this study**

To our knowledge, this study is the first in the UK to examine the non-random associations between diseases in a young, population-based cohort containing a high percentage of Black, Asian, and other ethnic minority groups. In addition, we compared clusters between different cohorts over time and found a high degree of similarity. We address the call of Whitty and Watt (2020) for a more generalized approach in the mapping of clusters.

**Implications of all the available evidence**

The links seen in previous studies between cardiometabolic diseases and chronic pain; cardiovascular diseases and dementia for older populations are supported in this study. In addition, mental health problems, risk factors and risky behaviours are the main concerns identified in a younger, multi-ethnic population.

Alt-text: Unlabelled box

## 1 Introduction

Multimorbidity, the co-occurrence of two or more diseases, is a growing global health challenge [1]. Multimorbidity increases in prevalence with age, with up to 95% of people aged 65 years and older showing clinical features [2]. Multimorbidity is strongly associated with social and material deprivation, and is a major driver of health care utilisation and mortality in deprived populations [3]. Recent research has focused on identifying the types of long term conditions (LTC) that cluster together in multimorbid patients [4]. This approach aims to identify which LTCs co-occur together more frequently to uncover new mechanisms of disease, offering clinicians the ability to develop multi-disease clinical strategies, avoiding conflicting treatment regimens and potential adverse drug effects and drug-drug interactions associated with polypharmacy [4], [5], [6].

Despite these advances, the concept and definition of multimorbidity remain elusive. The original concept of multimorbidity focused on the multiplicity of diseases but with little agreement on the set of diseases that were eligible for inclusion [7]. Advances in molecular medicine are revealing that our present understanding of nosology may be flawed because one molecular defect can result in several diseases and one disease may be associated with diverse molecular pathology [8]. The concept of multimorbidity has been extended to include not just diseases but ‘health conditions’ [1] including risk factors like hypertension, symptoms such as chronic pain, and measures of mental wellbeing including anxiety and depression [9]. Multimorbidity can be viewed as representing the intersection of multiple dimensions of poor health. The concept of intersectionality in health is commonly applied to the multiple overlapping social determinants that impact on the health of deprived and excluded populations [10], but the notion of intersectionality may be readily applicable in multimorbidity research. From a public health perspective, this approach to multimorbidity will contribute to understanding the social and environmental determinants of health and disease burden.

Most studies have identified multimorbidity as a manifestation of ageing, with age-associated frailty and multimorbidity being closely related concepts [11]. Urban populations, which account for 55% of the world population [12], are typically youthful. In Inner London, only 6·8% of the resident population is aged 65 years and over compared with 17·7% for England as a whole [13]. Urban environments are generally characterised by deprivation across multiple domains, a high proportion from ethnic minority and migrant populations, and by reduced life expectancy and life satisfaction compared with national comparators [13]. This research aimed to evaluate how multiple morbidity is expressed in an urban environment.

An initial approach to studying patterns of LTC combinations is to determine which conditions commonly co-occur together [9, [14], [15], [16]]. However, this approach tends to emphasise the most prevalent conditions, like hypertension, which are members of most high-frequency disease combinations [15]. It is more informative to view disease patterns in terms of relative frequencies through the evaluation of ‘associative multimorbidity’ [5]. Two systematic reviews [4, 5] revealed highly heterogeneous clusters of multimorbidity resulting, not only from the differing demographic characteristics of the sample populations, but also due to analytical clustering techniques employed. The aim of this study is to identify LTCs which tend to co-occur, in an inner-city primary care setting. Our objective was to find groups of conditions that are as correlated as possible among themselves and with as little correlation as possible with other groups in the data using Multiple correspondence analysis (MCA), a statistical technique to analyse clustering of multimorbidity [17], [18], [19].

## 2 Methods

### 2.1 Study design, setting and participants

The study was set in an inner-city borough in south London with a deprived, multi-ethnic, youthful population. In the UK, about 98% of the population is registered with a general practice. The population sample consisted of all patients registered at general practices in the borough (*n* = 41), except for patients (3·2%) who had opted out of anonymised data sharing for research. Anonymised coded data on all eligible patients aged 18 years and over between 1/4/2005 to 1/5/2020 were extracted from electronic health records (EHRs) held in primary care. The study is a retrospective cross-sectional on three continuous time periods from 2005 to 2020. For the purposes of clustering we did not consider the order of the conditions or the follow-up time, only which conditions occur together during each individual's period of registration. The proposal for the analysis of fully anonymised data was approved the by Lambeth Clinical Commissioning Group. Separate ethical committee approval was not required (Health Research Authority, personal correspondence) since all data were fully anonymised for the purposes of research access, and all patient identifiable data had been removed.

### 2.2 Data variables and measurement

Multimorbidity in this study is defined as the co-occurrence of two or more out of 32 LTCs, adapted from previous studies (Table S1) [16]. Nineteen LTCs, and the risk factors smoking, hypertension and obesity, were defined by the Quality and Outcomes Framework (QOF) [20]. The data codes and recording of these conditions are standardised nationally and recording rates are incentivised and therefore high. The rest were selected based on the importance within the urban, multi-ethnic community of our population sample. For these conditions we searched extensively for all possible Read (the clinical coding system used in UK general practice to record patient findings and procedures in health-care IT systems) and SNOMED codes that could represent each of these conditions, using the CPRD GOLD Codes List [21] as a starting point. Some conditions were defined by prescribing characteristics (such as ‘chronic pain’, defined by prescriptions of opioids); some were unlikely to result in medication (such as learning disability or morbid obesity). Patients were considered to have a LTC or multimorbidity if there is a record at any point in their adult life, and were included in analyses.

Demographic data consisted of gender, age in years at last known follow-up, and self-assigned ethnicity. Social deprivation data derived from participant postal code of residence were based on the Index of Multiple Deprivation (IMD) 2019 [22] classification at lower super output area, divided into quintiles based on the national distribution. The IMD is based on seven domains of deprivation including income, employment, education, health, crime, housing, and quality of living environment. Clinical data at last known follow-up included: the number of medications prescribed based on British National Formulary (BNF) sub-heading, risk of hospital admission in the next 12 months (based on the QAdmissions score > 20 [23]), and six risk factors based on whether a person was ever exposed: hypertension; moderate obesity (BMI 30·0–39·9 kg/m^2^), high cholesterol (total cholesterol > 5 mmol/L), smoking, elevated alcohol consumption (>14 units per week), and psychoactive substance use.

### 2.3 Statistical analysis

Sociodemographic, risk factor and clinical data were summarized for the multimorbid population using means and standard deviations, median and inter-quartile range (IQR), or counts and percentages as appropriate. Missing data were kept as missing.

MCA was carried out on the dataset where each condition was coded as present or absent. All individuals with multimorbidity were used as rows and binary LTCs as variables to determine principal dimensions. Age, gender, and number of conditions were used as supplementary variables (i.e. not used to determine principal dimensions, but with their coordinates plotted along with the LTCs). The number of dimensions considered for retention was based on the elbow of the scree plot.

The presence of each condition was mapped on a biplot, with the positions of the points on the map indicating positive association between conditions when they are close together or negative association when they are in opposite ends of the plot. To characterise each dimension, statistical parameters were calculated including the contribution of each LTC to the dimension and the representativeness of the dimension to the LTC using squared cosines (Table S2). These parameters were used to aid visual interpretation of the data, and to determine which conditions are redundant (do not contribute to patterns in the data) or highly relevant.

Variable coordinates derived from MCA were used to perform hierarchical clustering, using Ward's minimum variance method [24], to determine groups of co-morbid conditions. Overall, the number of clusters was determined based on the sum of squared errors, with the ratio of within sums of squares to between sums of squares presented to evaluate the distances of the clusters to each other. People were then assigned to clusters based on the proportion of their conditions that belong to a cluster (i.e. if more than 50% of a person's conditions belong to a particular cluster, then that person is deemed to belong to that cluster). Sociodemographic, risk factor and clinical data were summarised for each cluster.

### 2.4 Sensitivity analyses

The results of MCA and clustering were compared with those using Exploratory Factor Analysis on tetrachoric correlations using the principal axis factoring method. Due to the low prevalence of some of the LTCs, a frequency adjustment was applied that increased cells with a zero count to one-half. Factor dimensions were considered if each factor loading had at least two scores larger than 0·3 and the Heywood phenomenon was not observed. A second analyses was also performed to cluster all 32 conditions, not just the ones deemed well represented by MCA.

R version 4.0.2 and STATA were used for all analyses. This study is reported following *STROBE* guidelines for observational studies.

### 2.5 Role of the funding source

The funder had no role in the study design; in the collection, analysis, and interpretation of data; in the writing of the report; or in the decision to submit the paper for publication.

## 3 Results

### 3.1 Participants

The 41 practices participating in the study provided care to a population of 826,936 unique individuals aged 18 years and over, between 2005 and 2020; mean age 40 years (SD 15·6), 52% female, 54% white ethnicity, and 64% resident in socially deprived areas (two most deprived quintiles). Forty one percent of registered patients had at least one LTC and 21% (*n* = 174,881) had multimorbidity. Multimorbidity was more frequent among women (23%) than men (20%; χ^2^ *p* < 0.01). The number of conditions increased progressively with age, with those aged 80+ having a median of 5 (IQR=3) conditions compared with a median of 2 (IQR=1) conditions in those age 18–39 years.

### 3.2 Descriptive data

The characteristics of the multimorbid population are summarized in Table 1, stratified into three cohorts according to the year of last known follow-up (note that currently registered patients will belong in the most recent cohort). The most recent cohort has a higher prevalence of multimorbidity at 25% compared to 15–16% in the previous cohorts. All characteristics, apart from the proportion of substance use, change over time. The greatest changes were observed in the age and ethnicity structure: the 2016–2020 cohort has a younger age structure compared to those in 2005–2010, while Black and Asian ethnic groups feature prominently. Polypharmacy in the recent cohort decreases, while raised cholesterol and moderate obesity increases.

**Table 1:** Sociodemographic and clinical characteristics of the study sample (those with 2 or more long term conditions). Results are given as frequencies (column percent).

	2005–10	2011–15	2016–20	P value^a^
Number	25,052	27,528	122,301	
% of total population	15·5%	15·3%	25·2%	
Total Number of conditions				<0·001
2	11,012 (44·0)	12,322 (44·8)	52,192 (42·7)	
3	5827 (23·3)	6174 (22·4)	30,378 (24·8)	
≥4	8213 (32·8)	9032 (32·8)	39,731 (32·5)	
Female	13,747 (54·9)	15,141 (55·0)	68,150 (55·7)	0·004
Age at last known follow up				<0·001
18–39	7089 (28·3)	9677 (35·2)	38,148 (31·2)	
40–59	6050 (24·1)	7399 (26·9)	42,336 (34·6)	
60–79	6454 (25·8)	5773 (21·0)	30,435 (24·9)	
80+	5459 (21·8)	4679 (17·0)	11,382 (9·3)	
Recorded death at end of follow-up	8940 (35·7)	6900 (25·1)	6268 (5·1)	<0·001
Ethnicity				<0·001
White	12,181 (48·6)	17,248 (62·7)	68,988 (56·4)	
Black	2901 (11·6)	4335 (15·7)	29,090 (23·8)	
Asian	763 (3·0)	1423 (5·2)	7936 (6·5)	
Mixed	479 (1·9)	844 (3·1)	5571 (4·6)	
Other	274 (1·1)	396 (1·4)	2823 (2·3)	
Missing	8454 (33·7)	3282 (11·9)	7893 (6·5)	
IMD* quintile				0·042
1-most deprived	5086 (20·3)	5548 (20·2)	25,355 (20·7)	
2	11,360 (45·3)	12,550 (45·6)	56,226 (46·0)	
3	6576 (26·2)	7031 (25·5)	30,634 (25·0)	
4	1514 (6·0)	1706 (6·2)	7365 (6·0)	
5-least deprived	231 (0·9)	373 (1·4)	1372 (1·1)	
Missing	285 (1·1)	320 (1·2)	1349 (1·1)	
8 different medications in different BNF* sub-headings within last year	5783 (23·1)	5722 (20·8)	16,829 (13·8)	<0·001
>20% QAdmissions risk score	4 (0·0)	500 (1·8)	11,610 (9·5)	<0·001
Risk Factors				
Alcohol ≥ 14 units/week	177 (0·7)	653 (2·4)	2786 (2·3)	<0·001
Hypertension	9347 (37·3)	9162 (33·3)	40,786 (33·3)	<0·001
Cholesterol >5 mmol/L	8955 (35·7)	10,975 (39·9)	61,283 (50·1)	<0·001
BMI 30·0–39·9	4744 (18·9)	6549 (23·8)	41,677 (34·1)	<0·001
Current or ex-smoker	14,816 (59·1)	16,582 (60·2)	70,943 (58·0)	<0·001
Substance use	2225 (8·9)	2821 (10·2)	11,603 (9·5)	0·182

IMD = Index of Multiple Deprivation; BNF = British National Formulary.

a test for trend across periods.

### 3.3 Outcome data

**Fig. 1:**
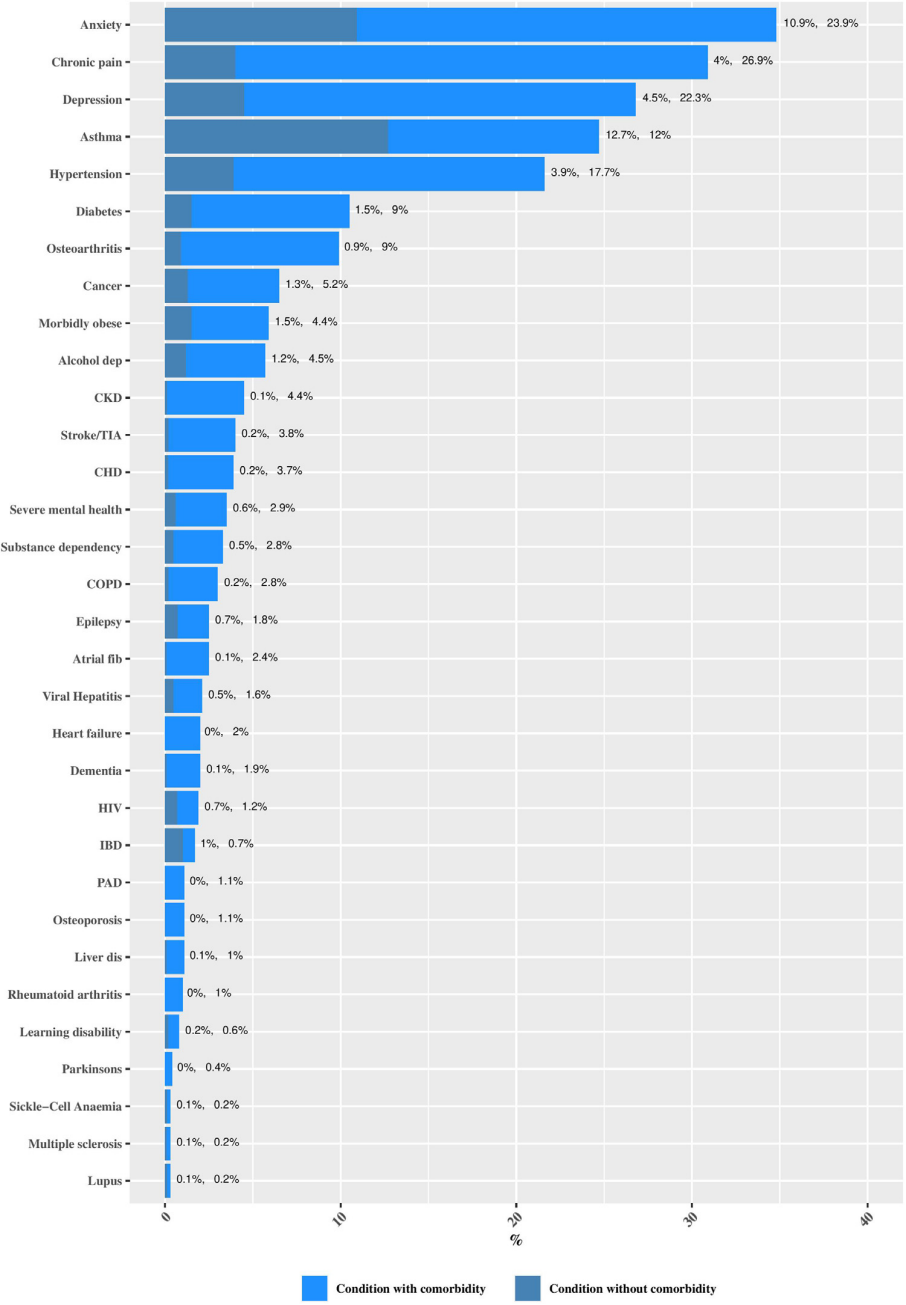
Prevalence of long-term conditions, with comorbidity and without comorbidity in those with one or more conditions (*n* = 335,863).

**Fig. 2:**
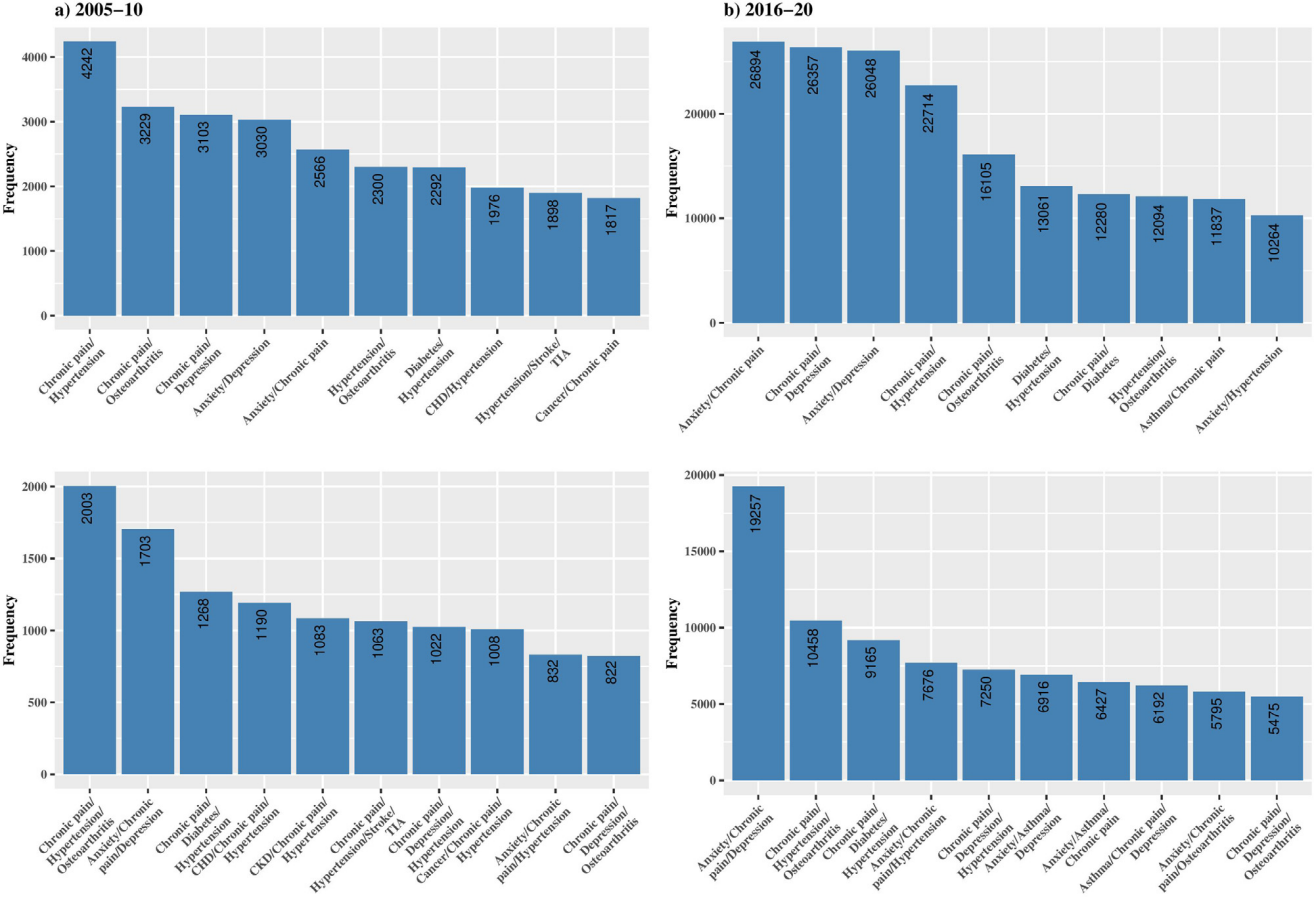
The ten most common dyads and triads in the multimorbid sample (*n* = 174,882), comparing the 2005–2010 vs. 2016–2020 cohort.

Anxiety (23·9%), chronic pain (26·9%), depression (22·3%), asthma (12%), hypertension (17·7%), diabetes (9%) and osteoarthritis (9%) make up the top seven most prevalent conditons (Fig. 1). Consequently, these conditions feature prominently in the most common dyads and triads (Fig. 2).

### 3.4 Main results

**Fig. 3:**
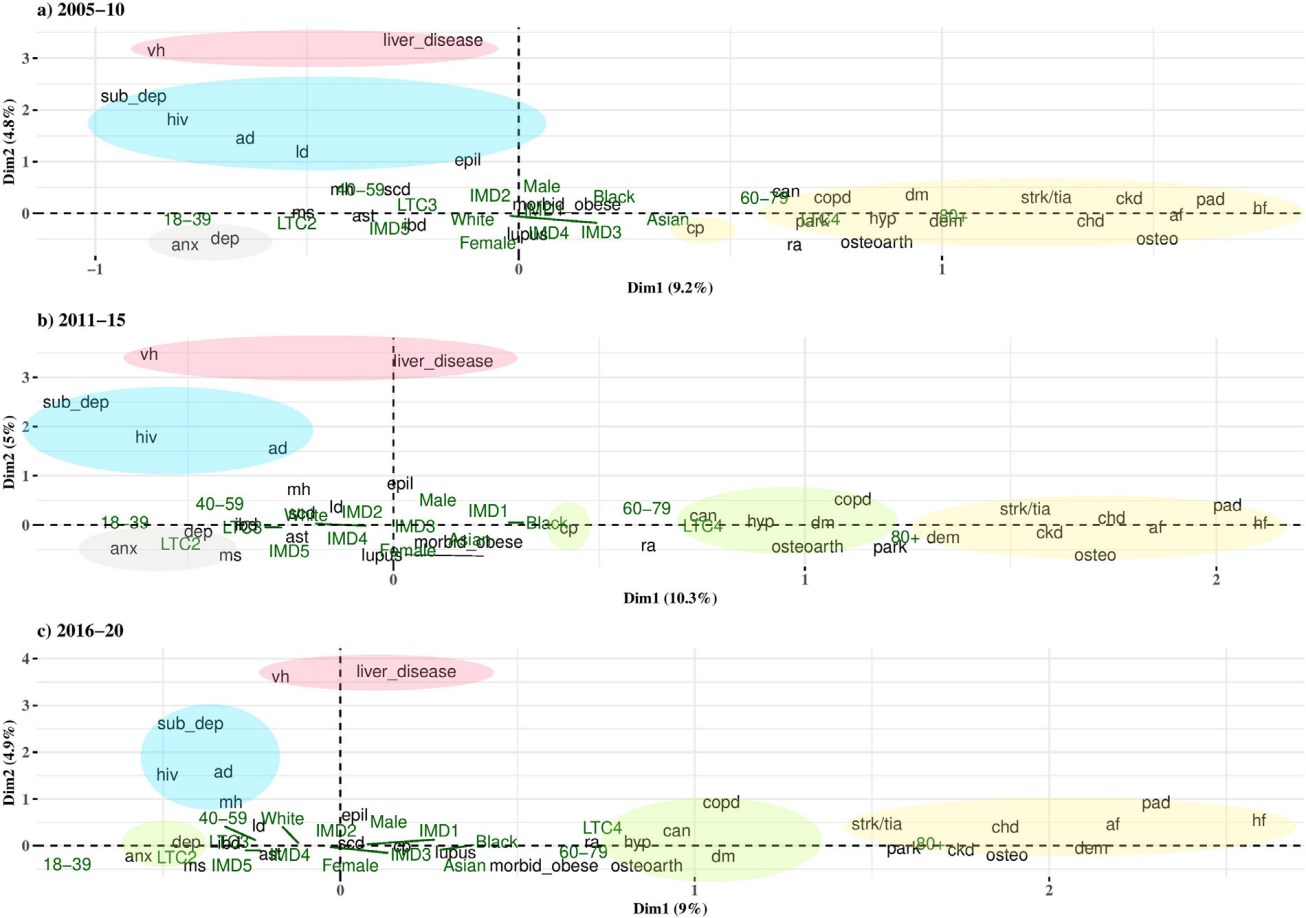
Graphical depiction of chronic diseases, age, gender, deprivation and ethnicity using Multiple Correspondence Analysis showing the projections on the plane defined by dimensions 1 and 2. The circles identifies clusters of conditions using hierarchical cluster analysis^a^ ^a^ad= Alcohol dependency, af = Atrial fibrillation, anx=Anxiety, ast=Asthma, can=Cancer, chd=Coronary heart disease, ckd=chronic kidney disease, copd= Chronic obstructive pulmonary disease, cp=Chronic pain, dem=Dementia, dep=Depression, dm=Type 2 diabetes, epil=Epilepsy, hf= Heart failure, hiv= human immunodeficiency virus, hyp=Hypertension",ibd= Inflammatory bowel disease, ld=Learning disability, mh=Severe mental health, ms=Multiple sclerosis, osteo=Osteoporosis, osteoarth=Osteoarthritis, pad= Peripheral artery disease, park=Parkinsons, ra=Rheumatoid arthritis, scd=Sickle-Cell Anaemia, strk/tia=Stroke/TIA, sub_dep=Substance dependency, vh=Viral hepatitis.

MCA retained 9·3% variance in the first dimension, and 4·8–5·0% in the second dimension (Fig. 3). The elbow of the scree plots (Fig. S1) suggested the retention of two dimensions; examination of dimensions three to 32 revealed only a small amount of information retained (4·2% to 1·6% of explained variance), therefore only the first two dimensions were considered further.

Fig. 3 presents the positions of LTCs in the first and second dimensions and the relationships between these conditions to supplementary variables and to each other. Similar patterns can be seen across time cohorts. The first dimension differentiates LTCs across age and number of conditions: anxiety, depression, substance dependency, viral hepatitis, HIV, and alcohol dependency are associated with a younger age and lower number of comorbidity (those with 2 LTCs), while stroke/transient ischaemic attack (TIA), chronic kidney disease (CKD), chronic heart disease (CHD), osteoporosis, PAD, atrial fibrillation, and heart failure are associated with the oldest age group. Conditions with a score close to one in the first dimension (i.e. COPD, diabetes, hypertension, osteoarthritis) are associated with high multimorbidity (≥4 LTCs), the 60–79 age group, and to Black and Asian ethnic groups. Gender and IMD score are closer to the centre of the plot, indicating a lower ability to discriminate between conditions.

Hierarchical cluster analysis was carried out on variables that are well represented by the first two dimensions of MCA (Table S1), and the individual clusters are identified in Fig. 3. The number of clusters was determined based on the loss of inertia and thus differs slightly across cohorts. However, the conditions which consistently occur together in the same cluster across cohorts are:

A) anxiety and depression (the “mental health” cluster); (Ratio of Within Sums of Squares(WSS)/Between Sums of Squares (BSS) ranged from <0·01 in 2005–10 to <0·01 in 2016–20)
B) heart failure, atrial fibrillation, CKD, CHD, stroke/TIA, PAD, dementia, and osteoporosis (the “cardiovascular” cluster); (WSS/BSS = 0·09–0·12)
C) osteoarthritis, cancer, chronic pain, hypertension, and diabetes (the “pain” cluster); (WSS/BSS = 0·05–0·06)
D) chronic liver disease and viral hepatitis (the “liver disease” cluster); (WSS/BSS = 0·02–0·03)
E) substance and alcohol dependency and HIV (the “dependence” cluster); (WSS/BSS = 0·37–0·55)

Some differences between cohorts include the appearance of epilepsy in cluster E (2005–10 cohort), and the appearance of severe mental health in cluster E (2016–20 cohort). A large difference is the combining of the mental health cluster A with chronic pain and the high prevalence cluster C in the 2016–20 cohort.

Table 2 presents frequencies for each condition at different levels of multimorbidity, across all patients. Cluster A shows a low multimorbidity burden – for example, of those diagnosed with anxiety (*n* = 80,284), 41% have only one other condition and 32% have 3 or more additional conditions. In contrast, conditions in Cluster B have a high multimorbidity burden – 86% of patients diagnosed with heart failure will also have 3 or more conditions. Conditions which were not considered for clustering are those that present early in childhood (e.g. asthma, sickle cell anaemia, learning disability).

**Table 2:** Multimorbidity associated with individual conditions. Results are given as frequencies (row percent).

LTC	Total	LTC+1 other condition	LTC+2 other conditions	LTC+3 or more conditions
	174,882	75,527	42,379	56,976
**Cluster A**				
Anxiety	80,284	33,293 (41·5)	21,201 (26·4)	25,790 (32·1)
Depression	74,930	27,286 (36·4)	20,411 (27·2)	27,233 (36·3)
**Cluster B**				
Heart failure	6788	352 (5·2)	580 (8·5)	5856 (86·3)
PAD	3576	176 (4·9)	320 (8·9)	3080 (86·1)
Osteoporosis	3634	213 (5·9)	365 (10)	3056 (84·1)
Atrial fibrillation	8178	723 (8·8)	927 (11·3)	6528 (79·8)
CHD	12,360	1121 (9·1)	1421 (11·5)	9818 (79·4)
CKD	14,726	1274 (8·7)	1853 (12·6)	11,599 (78·8)
Stroke/TIA	11,129	1145 (10·3)	1472 (13·2)	8512 (76·5)
Dementia	6342	507 (8)	812 (12·8)	5023 (79·2)
**Cluster C**				
Osteoarthritis	30,083	4247 (14·1)	5264 (17·5)	20,572 (68·4)
Cancer	17,511	3556 (20·3)	3521 (20·1)	10,434 (59·6)
Chronic pain	90,302	20,934 (23·2)	23,784 (26·3)	45,584 (50·5)
Hypertension	59,295	13,270 (22·4)	12,200 (20·6)	33,825 (57)
Diabetes	30,121	6180 (20·5)	5614 (18·6)	18,327 (60·8)
**Cluster D**				
Liver disease	3279	665 (20·3)	583 (17·8)	2031 (61·9)
Viral hepatitis	5302	1459 (27·5)	1116 (21)	2727 (51·4)
**Cluster E**				
Alcohol dep.	15,233	3611 (23·7)	3375 (22·2)	8247 (54·1)
Substance dep.	9437	2036 (21·6)	1979 (21)	5422 (57·5)
HIV	4101	1398 (34·1)	1013 (24·7)	1690 (41·2)
**No cluster**				
Asthma	40,299	15,775 (39.1)	9250 (23)	15,274 (37.9)
COPD	9276	849 (9.2)	1145 (12.3)	7282 (78.5)
Epilepsy	6091	1800 (29.6)	1284 (21.1)	3007 (49.4)
IBD	2247	1073 (47.8)	518 (23.1)	656 (29.2)
Learning disability	1875	580 (30.9)	474 (25.3)	821 (43.8)
Lupus	702	162 (23.1)	152 (21.7)	388 (55.3)
Severe mental health	9786	2324 (23.7)	2218 (22.7)	5244 (53.6)
Morbid obesity	14,762	3610 (24.5)	3004 (20.3)	8148 (55.2)
Multiple sclerosis	684	256 (37.4)	166 (24.3)	262 (38.3)
Parkinsons	1231	145 (11.8)	186 (15.1)	900 (73.1)
Rheumatoid arthritis	3444	747 (21.7)	684 (19.9)	2013 (58.4)
Sickle-Cell Anaemia	655	287 (43.8)	150 (22.9)	218 (33.3)

LTC = Long term condition, CHD=Coronary heart disease, CKD=chronic kidney disease, COPD= Chronic obstructive pulmonary disease, HIV= human immunodeficiency virus, IBD= Inflammatory bowel disease, PAD= Peripheral artery disease, TIA=Transient ischaemic attack.

Patients were grouped according to the clusters found based on their disease prevalence. As the analysis focused on the clusters of diseases rather than individuals, some people will have conditions that span across multiple clusters. For example, for persons assigned to cluster B, on average, 35% of their conditions could also belong to cluster C (Table S3). Reflecting the plots from MCA, those in cluster A, D and E tend to be younger (18–59), while cluster B tend to be older (80+; Table 3). Those in Cluster B and C have high cholesterol and obesity. The majority in Clusters D and E are males and current or past smokers (76%), while Black ethnicity features in Clusters C and D (32% and 26% in this cluster are of Black ethnicity, compared with 12–19% in other clusters). Clusters B and C have the highest number of medications. Deprivation does not seem to distinguish between clusters.

**Table 3:** Sociodemographic and clinical characteristics of clusters common to all time cohorts (2005–2020). Results are given as frequencies (column percent).

	A	B	C	D	E	F
	Mental health+	Cardiovascular+	Pain+	Liver+	Dependence+	Patients that do not belong to any cluster
**Number**	67,253	11,900	57,955	1369	3309	33,096
**Gender**						
Male	25,487 (37·9)	6725 (56·5)	26,118 (45·1)	948 (69·2)	2619 (79·1)	15,946 (48·2)
Female	41,765 (62·1)	5175 (43·5)	31,837 (54·9)	421 (30·8)	690 (20·9)	17,150 (51·8)
**Age**						
18–39	39,373 (58·5)	344 (2·9)	5065 (8·7)	516 (37·7)	1615 (48·8)	8001 (24·2)
40–59	22,728 (33·8)	1550 (13·0)	17,436 (30·1)	711 (51·9)	1488 (45·0)	11,872 (35·9)
60–79	4713 (7·0)	4168 (35·0)	24,502 (42·3)	138 (10·1)	203 (6·1)	8938 (27·0)
80+	439 (0·7)	5838 (49·1)	10,952 (18·9)	4 (0·3)	3 (0·1)	4285 (12·9)
**Ethnicity**						
White	43,966 (65·4)	6720 (56·5)	25,013 (43·2)	595 (43·5)	2121 (64·1)	20,002 (60·4)
Black	8531 (12·7)	2074 (17·4)	18,544 (32·0)	357 (26·1)	447 (13·5)	6374 (19·3)
Asian	2938 (4·4)	809 (6·8)	4710 (8·1)	125 (9·1)	83 (2·5)	1457 (4·4)
Mixed	3154 (4·7)	274 (2·3)	1923 (3·3)	55 (4·0)	141 (4·3)	1347 (4·1)
Other	1452 (2·2)	163 (1·4)	1273 (2·2)	25 (1·8)	50 (1·5)	530 (1·6)
**IMD quintile**						
1 - most deprived	11,589 (17·2)	2426 (20·4)	13,293 (22·9)	298 (21·8)	646 (19·5)	7737 (23·4)
2	31,091 (46·2)	5184 (43·6)	26,370 (45·5)	687 (50·2)	1655 (50·0)	15,150 (45·8)
3	18,180 (27·0)	3241 (27·2)	13,873 (23·9)	305 (22·3)	767 (23·2)	7875 (23·8)
4	4499 (6·7)	859 (7·2)	3406 (5·9)	53 (3·9)	136 (4·1)	1632 (4·9)
5 - least deprived	1038 (1·5)	102 (0·9)	496 (0·9)	12 (0·9)	30 (0·9)	298 (0·9)
**8 different medications in different BNF chapters within last year**	3587 (5·3)	3547 (29·8)	12,888 (22·2)	58 (4·2)	188 (5·7)	8066 (24·4)
**Alcohol ≥ 14 units**	1333 (2·0)	132 (1·1)	883 (1·5)	31 (2·3)	228 (6·9)	1009 (3·0)
**High cholesterol**	16,995 (25·3)	7441 (62·5)	38,577 (66·6)	264 (19·3)	715 (21·6)	17,221 (52·0)
**Hypertension**	3424 (5·1)	6959 (58·5)	38,087 (65·7)	52 (3·8)	65 (2·0)	10,708 (32·4)
**BMI 30·0–39·9**	11,331 (16·8)	3183 (26·7)	26,046 (44·9)	201 (14·7)	362 (10·9)	11,847 (35·8)
**Current or ex-smoker**	38,565 (57·3)	6737 (56·6)	30,722 (53·0)	757 (55·3)	2676 (80·9)	22,885 (69·1)
**Substance use**	5659 (8·4)	152 (1·3)	1778 (3·1)	298 (21·8)	2150 (65·0)	6612 (20·0)

The main cluster analyses were carried out on 20 of the 32 conditions that were well represented in MCA. We compared our results using EFA and a cluster analysis using all 32 conditions and found similar results. Fig. S2 shows conditions which are closest together on the tree are the ‘dependency and liver disease’, ‘ageing’ (heart conditions and dementia), anxiety and depression, hypertension and diabetes, chronic pain, cancer and osteoarthritis. The conditions identified in the main cluster analyses also load onto the same factor in EFA (Tables S4–S6). The exception is the loading of chronic pain onto a separate factor with rheumatoid arthritis, however in the MCA plots we see that these two conditions are located close to each other in the first dimension.

## 4 Discussion

### 4.1 Main findings

Using MCA to extract key patterns and discard noise from the data, this research found 20 conditions grouped together around five key clusters that remain consistent across time. The first dimension distinguishes conditions based on age and morbidity burden. In adults aged 18–39 years we identified the inter-connectedness of the highly prevalent conditions anxiety and depression diagnoses. A second cluster, associated with older age and polypharmacy, identified heart failure, PAD, osteoporosis, atrial fibrillation, CHD, CKD, stroke/TIA, and dementia as the most common co-occurring conditions. A third cluster, also occurring at older ages and particularly Black ethnic groups, connects highly prevalent conditions such as chronic pain, hypertension, diabetes mellitus and osteoarthritis. These conditions occur frequently in dyads and triads (Fig. 2) and are associated with high (≥4 LTC) multimorbidity. The second dimension identified the conditions HIV, viral hepatitis, liver disease, substance, and alcohol dependency, which predominantly occur in young males who are also likely to smoke. Social deprivation was not different across clusters, due to two possible reasons. Firstly, the variability between deprivation groups in this population is narrow, with just 1% in the least deprived national quintile. The small prevalence in this group makes it difficult to discriminate across conditions. Secondly, ethnicity is a stronger determinant, and this is often confounded by deprivation.

Across the whole population, the prevalence of multimorbidity increased over time, at 25% for the 2016–20 cohort compared to 15–16% for previous cohorts. The age and ethnicity structure of the multimorbid population changes to reflect a younger group with a higher proportion of Black and Asian ethnicities. The most prominent conditions in this population are not morbidities (physical diseases) but mental health conditions and chronic pain, as well as risk factors (hypertension, obesity, and alcohol).

### 4.2 Comparison with other studies

The finding of 21% prevalence of multimorbidity based on 32 conditions is comparable to studies reporting a similar number of conditions using an adult 18+ population [2, 9, 16]. Estimates of multimorbidity pattern prevalence differ in the literature because of variations in methods, data sources and structures, populations and LTCs studied. Although this makes it challenging to compare study results there are some similarities between the present and previous studies. For instance, the most common groups described in previous studies of multimorbidity patterns were cardiovascular and mental health. Clusters of high prevalence LTCs include cancer, hypertension, asthma, and depression [25], while clusters associated with high numbers of LTCs include hypertension, CHD, and diabetes. Clusters using older cohorts found clusters that are similar to our clusters B and C [19, 26]. Other studies which have established different comorbidity profiles by an index condition, have also determined a high multimorbidity burden in those with cardiovascular diseases, and a lower burden in people with mental health disorders [16, 17]. Substance misuse and mental illness clusters have been found to be more prevalent in younger, deprived communities [26], while cardiovascular multimorbidity has previously been shown to disproportionately affect black ethnic groups and women, and increases the likelihood of a diagnosis of chronic pain [27, 28].

### 4.3 Strengths and limitations

Due to the long time period (15 years) of data collection, this study performed MCA separately on 5-year cohorts, to see if different patterns emerged. Previous studies have limited analysis to currently registered patients, whereas this study includes ex-registered patients. As a result, we identified long-term stability in multimorbidity patterns. Only one cluster grouping changed substantially, with the emergence of a combined mental health cluster A with that of chronic pain and the high prevalence cluster C in the 2016–2020 cohort, which may reflect the increasing co-occurrence between mental and physical health issues [6, 26]. The number of determined clusters was based on the minimization of inertia, hence small changes in the groupings can be seen. However, the closeness of relationships, such as between anxiety and depression, between cardiovascular conditions, and the dependency/HIV and infectious disease conditions can be seen across all three cohorts, and this clinical consistency supported the analysis of clustering within the entire dataset.

This study benefits from having a large, relatively complete dataset. Selection of individual long term conditions and the definition of multimorbidity was identified using national guidelines as well as from discussions with local stakeholders, enabling identification of not only the ‘old age’ and ‘high morbidity/high prevalence’ clusters seen in other settings, but also clusters that are specific to this younger, multi-ethnic population, providing local relevance for service planning. However, the data only contains information on persons during their period of registration within a general practice, and we do not know what happens after they move away from the catchment area. The analysis does not consider the order of conditions, nor resolved conditions. Some conditions can be identified as resolved based on a standardised ruleset from the QOF (e.g. depression), but for non-QOF conditions (e.g. anxiety) ‘resolve’ codes are available but were not applied consistently. This means the relationship between anxiety and depression may change had coding of resolved conditions been more consistent. Relating to this, it is difficult to disentangle true population changes over time from increased data recording over time. Changes in LTC prevalence may be attributable to improved data recording, or true population changes.

### 4.4 What this study adds

This study not only identified clusters from the most prevalent conditions, but also clusters of conditions with low prevalence, which are missed when just examining the most common disease combinations in the form of dyads and triads - as the latter can be obscured by large data and may just be due to chance rather than identifying actual relationships [5]. Groups of conditions that remain consistent across time were identified, which is useful for future confirmatory research as well as to improve multimorbidity management. Analysis of a younger population identified a cluster of low prevalence conditions including dependency/HIV and infectious disease. The conditions in cluster D are known to co-occur, as Hepatitis B and C are common causes of chronic liver disease [29]. Similarly, people with alcohol and substance use disorders may be more at risk of HIV infection [30, 31].

This study highlighted gender differences in the rates of common mental disorders – anxiety and depression. These disorders, in which women predominate, affect approximately 1 in 5 people in the south London population and constitute a serious public health problem. Not only are these conditions diagnosed at a younger age, but as women tend to survive longer than men they will also live with multiple long-term conditions for longer [32]. Gender disparities seen in the dependency/HIV clusters are also prominent in our sample, with higher rates in men. The World Health Organization reports that in developed countries, the lifetime prevalence rate for substance and alcohol dependence is more than twice as high in men than women with 1 in 5 men vs 1 in 12 women developing dependence during their lives [32].

### 4.5 Implications

Multimorbidity research is now prioritizing identification of disease clusters. Mapping these clusters, and working out which are non-random, is crucial for three reasons: to uncover new mechanisms for disease; to develop treatments; and to reconfigure services to better meet patients’ needs [6]. With an ageing global population and a rise in disabling outcomes [33], it is necessary to continuously report on population health in detail, and to identify relationships between diseases to help decision makers identify ways of disease control and to better equip health services to deal with increasing burden of disease. Evidence suggests that multimorbidity prevalence is higher in urban versus rural areas [34]. Urbanisation is expected to increase, with many cities currently experiencing demographic changes with increasing migration and population densities. Thus, urban populations face challenges arising from increasing multimorbidity prevalence, severity, and complexity of conditions. This requires a tailored approach to care that considers these challenges, along with interventions designed to prevent and reduce avoidable disease burden [1]. For example, clinical management of HIV must consider possible diagnoses of co-morbid alcohol and substance use disorders or the possible prevention of these disorders [31]. Primary or secondary care that focusses on prevention of cardiovascular diseases, hypertension and diabetes is likely to delay the progression of severe multimorbidity in an ageing population.

### 4.6 Further work

Future work using this dataset will focus on the trajectories of diseases, to examine the onset of multimorbidity and their clustering. This analysis will take follow-up time into account, changing the study design to a longitudinal cohort study. We will use the clusters identified in this study to examine differences in patient consultation rates, and link to secondary care data to enable access to accurate hospital admissions and other important outcomes of multimorbidity.

### 4.7 Conclusion

This study has identified the co-morbidity between substance/alcohol dependency and HIV; liver disease and viral hepatitis; anxiety and depression; cardiometabolic diseases and chronic pain; heart conditions and dementia. These key relationships characterise the young urban population of south London. When considering interventions or medications for one condition, clinicians should account for the increased risk of the patient belonging to one cluster acquiring other LTCs within the same cluster.

## Author Contributions

**Alessandra Bisquera:** Conceptualization, Methodology, Software, Validation, Formal analysis, Writing - Original Draft, Writing - Review & Editing, Investigation, Visualisation, Project administration; **Martin Gulliford:** Writing - Original Draft, Writing - Review & Editing, Funding acquisition; **Hiten Dodhia**: Project administration, Conceptualization, Resources, Writing - Review & Editing, Investigation, Funding acquisition; **Lesedi Ledwaba-Chapman:** Validation, Formal analysis, Writing - Review & Editing; **Stevo Durbaba:** Data curation, Software; **Marina Soley-Bori:** Writing - Review & Editing; **Julia Fox-Rushby:** Writing - Review & Editing, Funding acquisition; **Mark Ashworth:** Conceptualization, Methodology, Resources, Writing - Review & Editing, Investigation, Supervision, Project administration, Funding acquisition; **Yanzhong Wang:** Conceptualization, Methodology, Resources, Writing - Review & Editing, Investigation, Supervision, Funding acquisition. All authors have verified the underlying data and accept responsibility to submit for publication.

## Data sharing

The data are not publicly available to share, but the research group can provide descriptive data in table form. Requests should be made to Mark Ashworth (mark.ashworth@kcl.ac.uk).

## Declaration of Interests

The authors declare no conflict of interest.

## Appendix Supplementary materials

Image, application 1

Image, application 2
