# Supplementary material for: Identifying longitudinal clusters of multimorbidity in an urban setting: A population-based cross-sectional study
Source: Lancet Reg Health Eur. 2021 Mar 2;3:100047. doi: 10.1016/j.lanepe.2021.100047 (PMC8454750; doi:10.1016/j.lanepe.2021.100047)
Supplement: Supplementary file 1 [file mmc1.pdf]

Supplementary tables and figures

Table S1 List of Long-Term Conditions (n = 32)

	**Inclusion in general practice quality and outcomes framework[a]**
Alcohol Dependence	No
Atrial Fibrillation	Yes
Anxiety Disorders	No
Asthma	Yes
Cancer	Yes
Chronic Kidney Disease (CKD) - Stages 3-5	Yes
Chronic obstructive pulmonary disease (COPD)	Yes
Chronic Pain (Chronic pain &/or analgesic opioid prescribing)	No
Coronary Heart Disease (CHD) (includes Angina, MI)	Yes
Dementia (includes Alzheimer's)	Yes
Depression	Yes
Diabetes mellitus	Yes
Epilepsy	Yes
Heart Failure	Yes
HIV/AIDS	No
Hypertension*	Yes
Inflammatory Bowel Disease (IBD)	No
Learning Disabilities	Yes
Liver Disease	No
Lupus	No
Serious Mental Illness	Yes
Morbid Obesity (BMI ≥40)	Yes
Multiple Sclerosis	No
Osteoporosis	Yes
Osteoarthritis	No
Peripheral Arterial Disease (PAD)	Yes
Parkinson's disease	No
Rheumatoid Arthritis	Yes
Sickle-Cell Anaemia	No
Substance dependency	No
Stroke and Transient Ischemic Attack (TIA)	Yes
Viral Hepatitis (B & C)	No

[a]The Quality and Outcomes Framework is a contractual arrangement for general practices that provides pay-for-performance incentives for LTC management

Justification of the use of Multiple Correspondence Analysis (MCA) and Hierarchical Cluster Analysis (HCA) to identify groups of long term conditions

MCA is a useful tool to uncover relationships between multiple categorical variables, by assessing relative frequencies in a similar fashion to that of a two-way contingency table for two binary variables. By using MCA, conditions were first screened for their discriminatory ability prior to clustering, to identify those which contribute highly to data variability. The key advantage of MCA is that it does not make any assumptions on the distribution of the data and requires no data transformations. It is possible to undertake this technique even on low prevalence variables. In contrast, Exploratory Factor Analysis requires data to be in a continuous format, which is unrealistic for diagnostic variables. Thus, most studies which use EFA on long term conditions requires the data to be transformed to tetrachoric correlations prior to analysis.[1]

Similarly, clustering techniques such as k-means and HCA is the most used technique to identify multimorbidity groups.[1] The advantage of hierarchical clustering is that it can accept many types of dissimilarity measures, including dimension scores from MCA, to identify clusters. Disadvantages are that it does not allow for conditions or patients to simultaneously belong to more than one cluster, and that it can be susceptible to outliers in the data, or the inclusion of inappropriate or irrelevant variables. We have tried to mitigate the last point by only including conditions which are shown to be 'relevant' in the first and second dimension in the clustering. Using both techniques is complementary – MCA is used to visualize relationships across all variables, and to identify variables that explain the most variation in the data, and HCA is used to cluster and identify groups to generate hypotheses in future studies.

MCA and cluster analysis are exploratory techniques and do not constitute hypothesis testing. The purpose in this study is to detect highly associated conditions in the data, using well established methods as well as clinical judgement. We have chosen to use the scree plot and other significance criterion such as the squared cosines to determine the number of dimensions and conditions to retain, however this means that conditions that were not relevant in the first or second dimensions were discarded. To counter this source of bias we have performed two sets of sensitivity analyses: the first considering all 32 conditions for the clustering analyses, and second using factor analyses with tetrachoric correlations, and found similar relationships between diseases in both methods.

Table S2 Percent contributions and squared cosines of each conditions for dimensions 1 and 2, derived from MCA. Highlighted values indicate contributions that are higher than expected, or good representation of that variable to the dimension

	2005-2010				2011-2015				2016-2020			
	Cos 1	Cos 2	%Contrib1	%Contrib2	Cos 1	Cos 2	%Contrib1	%Contrib2	Cos 1	Cos 2	%Contrib1	%Contrib2
Alcohol dep	0·0	0·2	1·2	14·1	0·0	0·3	0·3	14·6	0·0	0·3	0·2	14·4
Atrial fibrillation	0·2	0·0	5·5	0·0	0·2	0·0	6·0	0·0	0·2	0·0	6·2	0·3
Anxiety	0·3	0·2	6·0	7·0	0·3	0·1	4·4	3·8	0·3	0·0	4·5	0·5
Asthma	0·0	0·0	0·7	0·1	0·0	0·0	0·5	0·2	0·0	0·0	0·5	0·3
Cancer	0·1	0·0	1·8	0·4	0·1	0·0	2·4	0·1	0·1	0·0	2·4	0·2
CHD	0·3	0·0	8·3	0·0	0·3	0·0	7·8	0·0	0·2	0·0	7·2	0·4
CKD	0·2	0·0	7·2	0·0	0·3	0·0	9·0	0·0	0·3	0·0	8·6	0·0
COPD	0·1	0·0	1·7	0·1	0·1	0·0	2·3	0·6	0·1	0·1	2·0	3·1
Chronic pain	0·1	0·0	2·2	1·3	0·1	0·0	2·1	0·3	0·1	0·0	0·9	0·1
Dementia	0·1	0·0	1·8	0·0	0·1	0·0	3·5	0·1	0·1	0·0	3·9	0·0
Depression	0·3	0·1	5·4	3·9	0·2	0·0	3·7	1·4	0·2	0·0	3·7	0·0
Diabetes	0·2	0·0	4·5	0·8	0·2	0·0	4·8	0·1	0·2	0·0	6·5	0·4
Epilepsy	0·0	0·0	0·0	2·7	0·0	0·0	0·0	1·5	0·0	0·0	0·0	0·7
Heart failure	0·2	0·0	6·8	0·0	0·2	0·0	6·9	0·1	0·2	0·0	6·7	0·5
HIV	0·0	0·1	0·4	3·4	0·0	0·1	0·3	5·1	0·0	0·1	0·2	4·1
Hypertension	0·4	0·0	8·4	0·1	0·4	0·0	9·0	0·0	0·4	0·0	10·1	0·2
IBD	0·0	0·0	0·0	0·0	0·0	0·0	0·0	0·0	0·0	0·0	0·0	0·0
Learning disability	0·0	0·0	0·1	0·7	0·0	0·0	0·0	0·1	0·0	0·0	0·0	0·0
Liver disease	0·0	0·2	0·1	13·5	0·0	0·3	0·0	16·2	0·0	0·3	0·0	18·8
Lupus	0·0	0·0	0·0	0·1	0·0	0·0	0·0	0·1	0·0	0·0	0·0	0·0
Severe mental health	0·0	0·0	0·4	0·7	0·0	0·0	0·1	1·2	0·0	0·0	0·1	2·4
Morbidly obese	0·0	0·0	0·1	0·0	0·0	0·0	0·2	0·1	0·0	0·0	0·6	1·2
Multiple sclerosis	0·0	0·0	0·0	0·0	0·0	0·0	0·0	0·1	0·0	0·0	0·0	0·0
Osteoporosis	0·1	0·0	1·9	0·6	0·1	0·0	2·3	0·4	0·1	0·0	2·3	0·0
Osteoarthritis	0·2	0·0	5·0	1·1	0·2	0·0	5·5	0·4	0·2	0·0	6·0	0·5
PAD	0·1	0·0	3·2	0·1	0·1	0·0	3·2	0·1	0·1	0·0	2·7	0·7
Parkinson’s	0·0	0·0	0·3	0·0	0·0	0·0	0·4	0·1	0·0	0·0	0·5	0·0
Rheumatoid arthritis	0·0	0·0	0·4	0·5	0·0	0·0	0·3	0·2	0·0	0·0	0·3	0·1
Sickle-Cell Anaemia	0·0	0·0	0·0	0·1	0·0	0·0	0·0	0·0	0·0	0·0	0·0	0·0
Stroke/TIA	0·2	0·0	4·7	0·1	0·2	0·0	5·2	0·1	0·1	0·0	4·7	0·4
Substance dependency	0·1	0·3	2·0	17·0	0·0	0·3	0·9	19·5	0·0	0·3	0·5	20·4
Viral Hepatitis	0·0	0·3	0·7	20·6	0·0	0·4	0·3	25·1	0·0	0·4	0·1	24·9

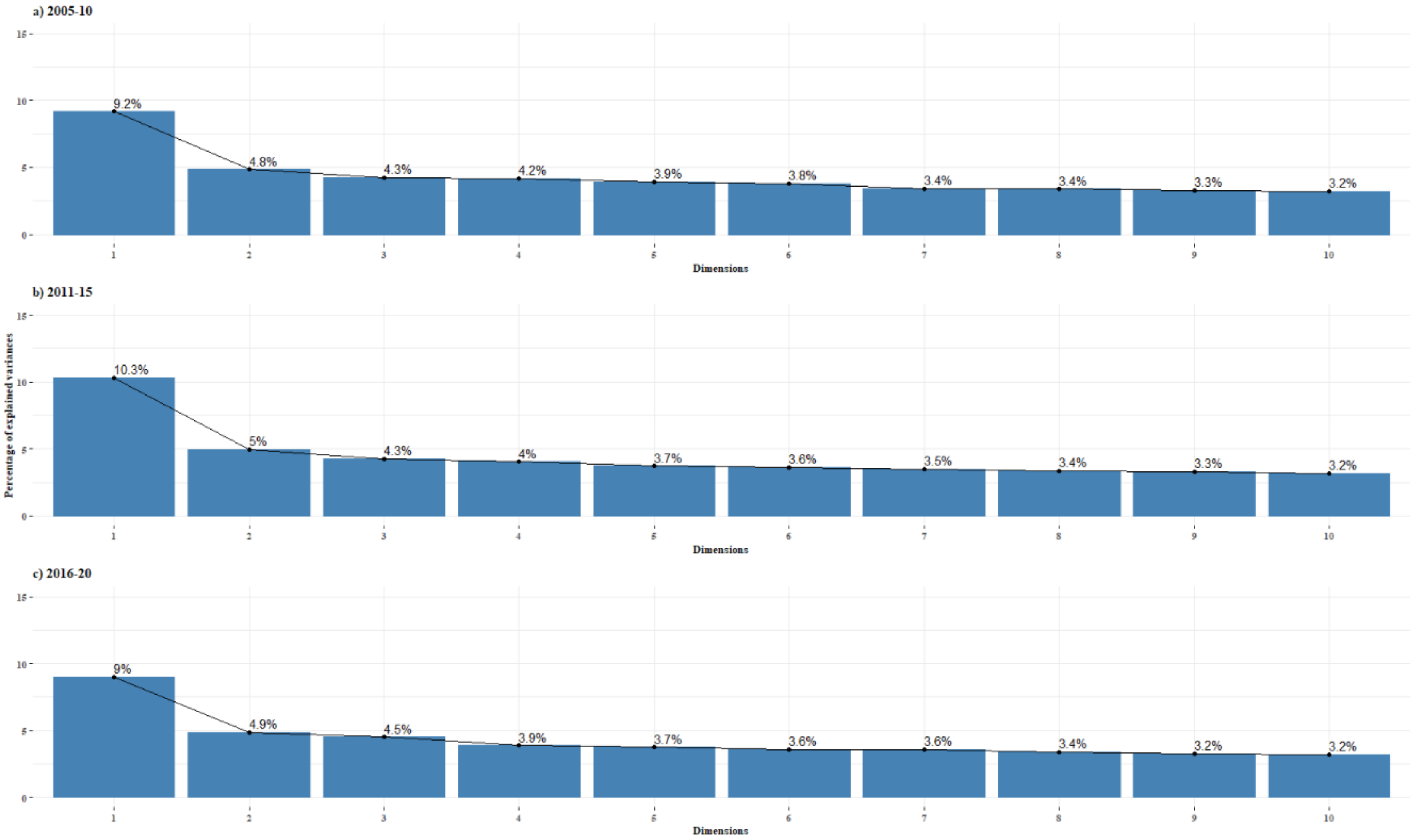

Fig S1: Scree plots from Multiple Correspondence Analysis

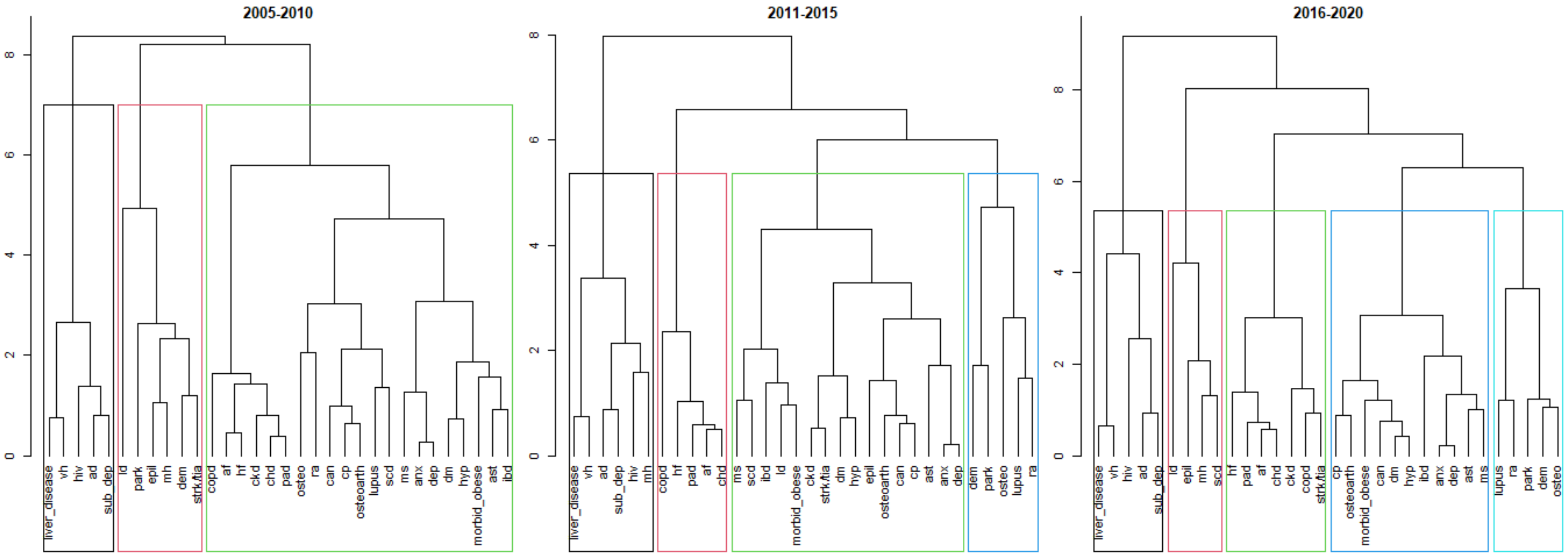

Fig S2: Hierarchical cluster analysis of all long-term conditions[a]

[a]ad= Alcohol dependency, af = Atrial fibrillation, anx=Anxiety, ast=Asthma , can=Cancer, chd=Coronary heart disease, ckd=chronic kidney disease, copd= Chronic obstructive pulmonary disease, cp=Chronic pain, dem=Dementia, dep=Depression, dm=Type 2 diabetes, epil=Epilepsy, hf= Heart failure , hiv= human immunodeficiency virus, hyp=Hypertension,ibd= Inflammatory bowel disease, ld=Learning disability, mh=Severe mental health, ms=Multiple sclerosis, osteo=Osteoporosis, osteoarth=Osteoarthritis, pad= Peripheral artery disease, park=Parkinsons, ra=Rheumatoid arthritis, scd=Sickle-Cell Anaemia, strk/tia=Stroke/TIA, sub_dep=Substance dependency, vh=Viral hepatitis

Table S3: The proportion of conditions that belong to each identified cluster. Individuals were assigned to a cluster if more than 50% of their conditions belong that cluster. Results are given as the mean proportion (sd)

Number of conditions that belong to the cluster divided by the total number of conditions for each person.	**Patients assigned to each cluster**				
	**A**	**B**	**C**	**D**	**E**
	67253	11900	57955	1369	3309
A	0.66 (0.20)	0.02 (0.06)	0.06 (0.11)	0.02 (0.07)	0.06 (0.11)
B	0.01 (0.05)	0.57 (0.12)	0.07 (0.12)	0.00 (0.03)	0.00 (0.03)
C	0.18 (0.21)	0.35 (0.17)	0.70 (0.20)	0.04 (0.10)	0.04 (0.09)
D	0.00 (0.04)	0.00 (0.03)	0.01 (0.06)	0.64 (0.21)	0.02 (0.08)
E	0.03 (0.11)	0.01 (0.06)	0.02 (0.09)	0.20 (0.24)	0.59 (0.16)

Table S4: Exploratory factor analysis of long-term conditions for the 2005-2010 cohort. Grey highlighted values show the LTCs which were used in the main cluster analysis, and the highest loadings onto a factor.

	Factor 1	Factor 2	Factor 3	Factor 4
Anxiety	-0·69	-0·21	-0·34	-0·21
Depression	-0·61	-0·20	-0·26	-0·37
Heart Failure	0·65	-0·11	-0·14	-0·16
PAD	0·52	0·02	-0·28	-0·03
Osteoporosis	0·33	-0·22	-0·32	0·13
Atrial Fibrillation	0·58	-0·13	-0·13	-0·09
CHD	0·65	-0·13	-0·23	-0·13
CKD	0·62	-0·15	-0·01	0·05
Stroke/TIA	0·58	-0·12	0·09	-0·08
Dementia	0·40	-0·33	0·17	-0·24
Osteoarthritis	0·37	-0·21	-0·24	0·26
Cancer	0·37	0·17	-0·17	0·06
Chronic Pain	0·17	-0·13	-0·26	0·67
Hypertension	0·71	-0·10	-0·07	-0·01
Diabetes	0·53	0·02	0·05	-0·05
Liver Disease	0·23	0·73	-0·01	-0·05
Viral Hepatitis	-0·04	0·84	-0·02	0·02
Alcohol Dependence	0·02	0·59	0·02	-0·36
Substance Misuse	-0·25	0·66	-0·05	-0·12
HIV	-0·23	0·44	0·13	-0·03
COPD	0·37	0·03	-0·21	-0·11
Learning Difficulties	-0·06	-0·12	0·82	-0·06
Epilepsy	0·08	0·09	0·64	0·01
Serious Mental Health	-0·12	-0·10	0·44	-0·24
Parkinson's	0·19	-0·50	0·38	-0·17
Sickle Cell Disease	-0·18	0·09	0·30	0·55
Lupus	-0·23	-0·14	0·09	0·62
Rheumatoid Arthritis	0·05	-0·09	-0·25	0·60
Asthma	-0·28	-0·03	-0·14	0·00
IBD	-0·20	-0·16	-0·07	-0·11
Morbid Obesity	0·02	-0·12	0·23	0·13
Multiple Sclerosis	-0·31	-0·11	0·10	0·14

Table S5: Exploratory factor analysis of long-term conditions for the 2011-2015 cohort. Grey highlighted values show the LTCs which were used in the main cluster analysis, and their highest loadings onto a factor.

	Factor 1	Factor 2	Factor 3	Factor 4	Factor 5
Anxiety	-0·41	-0·22	-0·27	0·52	-0·26
Depression	-0·37	-0·17	-0·12	0·55	-0·20
Heart Failure	0·74	-0·02	0·01	-0·08	-0·13
PAD	0·67	0·01	0·05	0·17	0·00
Osteoporosis	0·32	0·51	-0·16	0·23	-0·13
Atrial Fibrillation	0·71	0·03	-0·04	0·10	-0·07
CHD	0·76	0·01	-0·01	0·10	-0·04
CKD	0·74	0·03	-0·11	-0·08	-0·04
Stroke/TIA	0·58	0·11	-0·01	0·06	0·21
Dementia	0·41	0·29	-0·14	0·23	0·27
Osteoarthritis	0·43	0·38	-0·08	0·02	-0·09
Cancer	0·35	0·17	0·08	-0·05	0·00
Chronic Pain	0·04	0·85	-0·02	-0·03	-0·01
Hypertension	0·78	0·03	-0·10	-0·23	-0·08
Diabetes	0·58	-0·19	-0·07	-0·33	0·13
Liver Disease	0·22	-0·01	0·75	-0·06	-0·03
Viral Hepatitis	-0·14	-0·04	0·81	-0·24	-0·13
Alcohol Dependence	0·05	-0·09	0·64	0·38	0·19
Substance Misuse	-0·24	0·01	0·70	0·21	0·10
HIV	-0·23	-0·14	0·45	-0·25	-0·24
COPD	0·45	0·12	0·13	0·27	0·04
Rheumatoid Arthritis	-0·17	0·74	-0·04	-0·14	-0·07
Lupus	-0·23	0·37	-0·21	-0·24	0·01
Learning Difficulties	-0·09	-0·22	-0·16	-0·13	0·79
Epilepsy	-0·16	0·16	0·16	0·06	0·70
Serious Mental Health	-0·06	-0·06	0·08	0·08	0·35
Asthma	-0·10	-0·24	-0·15	0·11	-0·01
IBD	-0·06	-0·28	-0·09	-0·23	-0·10
Morbid Obesity	0·15	-0·31	-0·18	-0·40	0·11
Multiple Sclerosis	-0·38	0·14	-0·29	-0·18	0·10
Parkinson's	0·19	0·23	-0·17	0·18	0·22
Sickle Cell Disease	-0·33	0·11	-0·04	-0·53	-0·07

Table S6: Exploratory factor analysis of long-term conditions for the 2016-2020 cohort. Grey highlighted values show the LTCs which were used in the main cluster analysis, and their highest loadings onto a factor.

	Factor 1	Factor 2	Factor 3	Factor 4
Anxiety	-0·51	-0·18	-0·02	-0·54
Depression	-0·43	0·01	0·09	-0·57
Heart Failure	0·71	0·03	0·05	-0·01
PAD	0·61	0·15	0·10	-0·21
Osteoporosis	0·45	-0·06	0·41	-0·13
Atrial Fibrillation	0·67	-0·02	0·10	-0·07
CHD	0·72	0·04	0·06	-0·14
CKD	0·71	-0·08	0·01	0·04
Stroke/TIA	0·51	0·08	0·23	0·16
Dementia	0·55	-0·05	0·28	-0·03
Osteoarthritis	0·51	-0·13	0·22	-0·07
Cancer	0·42	0·08	0·04	-0·05
Chronic Pain	0·10	-0·07	0·76	0·02
Hypertension	0·82	-0·10	-0·14	0·07
Diabetes	0·62	-0·15	-0·30	0·13
Liver Disease	0·15	0·70	-0·04	0·13
Viral Hepatitis	0·01	0·79	-0·17	0·11
Alcohol Dependence	-0·05	0·64	0·06	-0·16
Substance Misuse	-0·20	0·73	0·09	-0·05
HIV	-0·05	0·44	-0·34	-0·10
COPD	0·41	0·32	0·28	-0·16
Parkinson's	0·36	-0·07	0·18	-0·06
Rheumatoid Arthritis	0·02	-0·10	0·60	0·14
Lupus	-0·08	-0·09	0·39	0·30
Epilepsy	-0·18	0·18	0·32	0·50
Learning Difficulties	-0·26	-0·03	0·04	0·67
Sickle Cell Disease	-0·19	-0·04	0·07	0·51
Asthma	-0·19	-0·13	-0·09	0·01
IBD	-0·09	-0·11	-0·21	0·13
Morbid Obesity	0·16	-0·25	-0·30	0·29
Multiple Sclerosis	-0·23	-0·23	0·17	-0·01
Serious Mental Health	-0·12	0·18	0·01	0·12

References

1. Prados-Torres A, Calderón-Larrañaga A, Hancco-Saavedra J, Poblador-Plou B, van den Akker M. Multimorbidity patterns: A systematic review. *J Clin Epidemiol*. 2014; **67**(3):254-66.
